# *Vital Signs:* Estimated Proportion of Adult Health Problems Attributable to Adverse Childhood Experiences and Implications for Prevention — 25 States, 2015–2017

**DOI:** 10.15585/mmwr.mm6844e1

**Published:** 2019-11-08

**Authors:** Melissa T. Merrick, Derek C. Ford, Katie A. Ports, Angie S. Guinn, Jieru Chen, Joanne Klevens, Marilyn Metzler, Christopher M. Jones, Thomas R. Simon, Valerie M. Daniel, Phyllis Ottley, James A. Mercy

**Affiliations:** ^1^Division of Violence Prevention, National Center for Injury Prevention and Control, CDC; ^2^Division of Injury Prevention, National Center for Injury Prevention and Control, CDC; ^3^National Center for Injury Prevention and Control, CDC.

## Abstract

**Introduction:**

Adverse childhood experiences, such as violence victimization, substance misuse in the household, or witnessing intimate partner violence, have been linked to leading causes of adult morbidity and mortality. Therefore, reducing adverse childhood experiences is critical to avoiding multiple negative health and socioeconomic outcomes in adulthood.

**Methods:**

Behavioral Risk Factor Surveillance System data were collected from 25 states that included state-added adverse childhood experience items during 2015–2017. Outcomes were self-reported status for coronary heart disease, stroke, asthma, chronic obstructive pulmonary disease, cancer (excluding skin cancer), kidney disease, diabetes, depression, overweight or obesity, current smoking, heavy drinking, less than high school completion, unemployment, and lack of health insurance. Logistic regression modeling adjusting for age group, race/ethnicity, and sex was used to calculate population attributable fractions representing the potential reduction in outcomes associated with preventing adverse childhood experiences.

**Results:**

Nearly one in six adults in the study population (15.6%) reported four or more types of adverse childhood experiences. Adverse childhood experiences were significantly associated with poorer health outcomes, health risk behaviors, and socioeconomic challenges. Potential percentage reductions in the number of observed cases as indicated by population attributable fractions ranged from 1.7% for overweight or obesity to 23.9% for heavy drinking, 27.0% for chronic obstructive pulmonary disease, and 44.1% for depression.

**Conclusions and implications for public health practice:**

Efforts that prevent adverse childhood experiences could also potentially prevent adult chronic conditions, depression, health risk behaviors, and negative socioeconomic outcomes. States can use comprehensive public health approaches derived from the best available evidence to prevent childhood adversity before it begins. By creating the conditions for healthy communities and focusing on primary prevention, it is possible to reduce risk for adverse childhood experiences while also mitigating consequences for those already affected by these experiences.

## Introduction

Healthy child development contributes to overall population health and prosperity. Decades of research have shown that exposure to violence in childhood (e.g., physical, sexual, or psychological) and witnessing potentially traumatic experiences in the home (e.g., intimate partner violence, mental illness, or substance misuse), collectively referred to as adverse childhood experiences, can have profound and lasting negative effects on health and social outcomes (*1*–*8*). Given the connection between adverse childhood experiences and health, preventing these experiences is strategic for reducing several of the leading causes of adult morbidity and mortality.

Adverse childhood experiences are common and have important implications for health and well-being (*6*,*9*). Whereas everyone is at risk for adverse childhood experiences, numerous studies have documented inequities in such experiences attributed to the historical, social, and economic environments in which some families live (*9*–*11*).

Exposure to adverse childhood experiences can be traumatic, evoking toxic stress responses that have immediate and long-term adverse physiologic and psychologic impacts. These adverse childhood experiences can derail optimal health and development by altering gene expression, brain connectivity and function, immune system function, and organ function (*8*). Adverse childhood experiences can also compromise development of healthy coping strategies, which can affect health behaviors, physical and mental health, life opportunities, and premature death (*1*–*8*,*12*). Adverse childhood experiences have been linked to increased risk for alcohol and substance use disorders, suicide, mental health conditions, heart disease, other chronic illnesses, and health risk behaviors throughout life. Adverse childhood experiences have also been linked to reduced educational attainment, employment, and income, which directly and indirectly affect health and well-being (*1*–*8*). At least five of the 10 leading causes of death have been associated with exposure to adverse childhood experiences, including several contributors to declines in life expectancy (*6*,*13*).

Adverse childhood experiences are preventable (*14*–*16*). Randomized controlled and matched-group trials have demonstrated 48%–52% reductions in rates of child abuse and neglect associated with preschool enrichment and early childhood home visitation programs (*14*,*15*). Preventing adverse childhood experiences is critical to addressing multiple public health and social challenges and to improving the lives of children, families, and communities. To understand the potential impact of preventing adverse childhood experiences in reducing negative health and well-being outcomes, state survey data were used to estimate population attributable fractions representing potential percentage reductions in the number of observed cases of health conditions, health risk behaviors, and socioeconomic impacts.

## Methods

The Behavioral Risk Factor Surveillance System (BRFSS)* is a state-based telephone survey of noninstitutionalized adults administered annually within each state, the District of Columbia, and U.S. territories. Participants report on a range of health conditions and risk behaviors. During the 2015–2017 data collection years, 27 states included state-added adverse childhood experience questions, in addition to the standardized set of BRFSS questions. These 11 state-added questions assess exposure to eight types of adverse childhood experiences: three types of abuse (physical, emotional, and sexual) and five types of household challenges (household member substance misuse, incarceration, mental illness, parental divorce, or witnessing intimate partner violence) before age 18 years. The adverse childhood experience items administered on the California and New Hampshire BRFSS surveys were inconsistent with those administered by the other states and were excluded, leaving 25 states^†^ in these analyses. Data were collected from 144,017 respondents who answered all adverse childhood experience questions and provided responses for age, race/ethnicity, and sex. Each respondent was classified into one of the following adverse childhood experience exposure categories based on the number of adverse childhood experience types reported: zero, one, two or three, and four or more types of adverse childhood experience exposure. The content and scoring of BRFSS adverse childhood experience items have been previously described (*17*).

Associations between outcomes and adverse childhood experience exposure were assessed. Coronary heart disease, stroke, asthma, chronic obstruction pulmonary disease (COPD), cancer (excluding skin cancer), kidney disease, diabetes, and depression were measured by asking respondents whether they had ever been told by a health care professional that they had the condition. Body mass index (BMI), calculated from self-reported height and weight, was used to determine each participant’s overweight or obesity status (overweight defined as BMI of ≥25 kg/m^2^; obesity defined as BMI of ≥30 kg/m^2^). Current smoking was defined as lifetime smoking of at least 100 cigarettes and currently smoking on at least some days. Heavy drinking was defined as adult men consuming at least 15 alcoholic beverages per week or adult women consuming at least eight alcoholic beverages per week in the past 30 days. Socioeconomic challenges included current lack of health insurance, current unemployment status, and attainment of less than a high school diploma or equivalent education.

As a preliminary step, the frequency distributions, including weighted percentages and corresponding 95% confidence intervals (CIs) of adverse childhood experience exposure by sociodemographic characteristics, were estimated. The overall bivariate associations between adverse childhood experience score and each sociodemographic variable were subsequently tested using chi-squared tests of independence. Logistic regression models were used to quantify the associations between adverse childhood experience exposure and each of the health outcomes, health risk behaviors, and socioeconomic challenges. All models were adjusted for race/ethnicity (non-Hispanic white [white], non-Hispanic black [black], non-Hispanic American Indian/Alaska Native [AI/AN], non-Hispanic Asian [Asian], Hispanic, and non-Hispanic other [Other]^§^); sex (male or female); and age group (18–24, 25–34, 35–44, 45–54, 55–64, and ≥65 years). Population attributable fractions, adjusted for age, race/ethnicity, and sex, were estimated using the predicted probabilities from the models to ascertain the percentage reduction in the number of observed cases of each outcome that would be expected if adverse childhood experience exposure were incrementally reduced or eliminated in the study population (*18*). R (version 3.6.0; R Core Team) was used for all analyses and accounted for the complex survey design. Response rates for the states analyzed ranged from 30.6% to 59.0%.

## Results

Overall, 60.9% of adults in the study population experienced at least one type of adverse childhood experience, and 15.6% experienced four or more types (Table 1). Sex, race/ethnicity, and age group were independently associated with adverse childhood experience exposure. Women, AI/AN, blacks, and the Other racial/ethnic group were more likely to experience four or more types of adverse childhood experiences than were men and whites. Younger adults reported exposure to more adverse childhood experience types than did older adults, particularly those aged ≥65 years.

**TABLE 1:** Sociodemographic characteristics of adults in the study population by adverse childhood experience score* — Behavioral Risk Factor Surveillance System (BRFSS), 25 states,^†^ 2015–2017

Characteristic	Adverse childhood experience score							
	0		1		2–3		≥4	
	No.	%^§^ (95% CI)	No.	%^§^ (95% CI)	No.	%^§^ (95% CI)	No.	%^§^ (95% CI)
**Sex^¶^**								
Men	26,852	39.3 (38.5–40.0)	14,590	24.7 (24.0–25.3)	12,340	22.2 (21.5–22.8)	6,781	13.9 (13.4–14.5)
Women	36,513	38.8 (38.2–39.5)	18,570	22.3 (21.7–22.9)	16,802	21.7 (21.1–22.3)	11,569	17.1 (16.6–17.7)
**Age group (yrs)^¶^**								
18–24	2,178	29.5 (27.7–31.3)	1,763	24.3 (22.6–25.9)	1,768	25.0 (23.4–26.7)	1,456	21.2 (19.6–22.7)
25–34	3,961	30.5 (29.2–31.9)	2,878	22.9 (21.6–24.2)	3,030	24.8 (23.5–26.1)	2,654	21.8 (20.5–23.1)
35–44	5,617	35.0 (33.7–36.4)	3,711	23.1 (21.9–24.3)	3,663	23.1 (22.0–24.3)	2,998	18.7 (17.7–19.8)
45–54	8,797	37.5 (36.3–38.7)	5,332	23.5 (22.5–24.5)	5,206	22.9 (21.9–24.0)	3,685	16.1 (15.2–17.0)
55–64	13,984	41.4 (40.4–42.5)	7,451	23.3 (22.3–24.2)	6,883	21.6 (20.7–22.4)	4,099	13.7 (13.0–14.5)
≥65	28,828	52.1 (51.3–53.0)	12,025	23.7 (23.0–24.5)	8,592	16.9 (16.2–17.5)	3,458	7.3 (6.8–7.7)
Race/Ethnicity^¶,^**								
White	52,614	40.2 (39.7–40.7)	26,451	23.1 (22.7–23.6)	22,855	21.7 (21.2–22.2)	13,934	15.0 (14.6–15.4)
Black	4,591	32.0 (30.5–33.5)	3,209	26.4 (24.9–27.8)	2,782	24.0 (22.6–25.4)	1,498	17.7 (16.3–19.0)
American Indian/Alaska Native	838	28.8 (24.6–32.9)	588	21.2 (17.2–25.3)	677	21.6 (17.3–25.9)	726	28.3 (24.1–32.6)
Asian	1,038	56.3 (52.5–60.1)	350	19.8 (16.8–22.8)	283	15.3 (12.7–17.9)	116	8.6 (5.9–11.2)
Hispanic	3,434	38.2 (36.3–40.1)	1,953	23.2 (21.6–24.9)	1,891	22.7 (21.1–24.3)	1,349	15.8 (14.5–17.2)
Other	850	25.5 (22.1–28.9)	609	24.2 (20.7–27.7)	654	22.3 (19.4–25.1)	727	28.0 (24.7–31.4)
**Total**	**63,365**	**39.0 (38.6–39.5)**	**33,160**	**23.4 (23.0–23.9)**	**29,142**	**21.9 (21.5–22.4)**	**18,350**	**15.6 (15.2–16.0)**

**Abbreviation:** CI = confidence interval.

* Based on the number of adverse childhood experience types reported.

^†^ States with state-added adverse childhood experience questions: Alaska, Kansas, Kentucky, Maryland, Ohio, South Carolina, and Texas (2015); Arizona, Arkansas, Georgia, Louisiana, Michigan, New York, Oklahoma, Pennsylvania, and Utah (2016); Connecticut, Illinois, Iowa, Nevada, Oregon, South Dakota, Tennessee, Virginia, and Wisconsin (2017).

**^§^** Percentages are weighted estimates; analyzed data are from 25 states with state-added adverse childhood experience questions on BRFSS.

**^¶^** p<0.001 from chi-squared test of independence.

** Participants self-reporting as white, black, American Indian/Alaska Native, Asian, and Other (Native Hawaiian or Other Pacific Islander, multiracial, or other) were non-Hispanic; Hispanic participants could be of any race.

Logistic regression analysis of the association between adverse childhood experience exposure and the health outcomes examined found that adults with the highest level of adverse childhood experience exposure had higher odds of having chronic health conditions, with adjusted odds ratios (AORs) ranging from 1.2 (95% CI = 1.1–1.3) for overweight or obesity to 2.8 (95% CI = 2.5–3.1) for COPD, compared with those reporting no adverse childhood experience exposure (Table 2). After adjusting for age, sex, and race/ethnicity, odds of depression (AOR = 5.3, 95% CI = 4.9–5.7), being a current smoker (AOR = 3.1, 95% CI = 2.8–3.3) or heavy drinker (AOR = 1.8, 95% CI = 1.6–2.0), and socioeconomic challenges including current unemployment (AOR = 1.7, 95% CI = 1.5–2.0) were also higher among adults with the highest levels of adverse childhood experience exposure, compared with those reporting no adverse childhood experience exposure.

**TABLE 2:** Association between adverse childhood experience score*^,†^ and health conditions, health risk behaviors, and socioeconomic challenges — Behavioral Risk Factor Surveillance System, 25 states,^§^ 2015–2017

Outcome	Adverse childhood experience score		
	1	2–3	≥4
	Adjusted odds ratio (95% CI)		
**Chronic condition**			
Coronary heart disease	1.1 (1.0–1.3)	1.2 (1.1–1.4)	1.8 (1.6–2.1)
Stroke	1.1 (1.0–1.3)	1.3 (1.2–1.5)	2.1 (1.7–2.5)
Asthma	1.3 (1.2–1.4)	1.6 (1.4–1.7)	2.2 (2.0–2.4)
Chronic obstructive pulmonary disease	1.3 (1.1–1.4)	1.7 (1.5–1.9)	2.8 (2.5–3.1)
Cancer (excluding skin)	1.1 (1.0–1.1)	1.2 (1.1–1.3)	1.4 (1.2–1.6)
Kidney disease	1.2 (1.0–1.4)	1.3 (1.2–1.6)	1.7 (1.4–2.0)
Diabetes	1.0 (0.9–1.1)	1.1 (1.1–1.2)	1.4 (1.2–1.5)
Overweight or obesity^¶^	1.0 (0.9–1.1)	1.1 (1.0–1.2)	1.2 (1.1–1.3)
**Mental health**			
Depression	1.6 (1.5–1.7)	2.6 (2.4–2.8)	5.3 (4.9–5.7)
**Health risk behavior**			
Current smoker	1.4 (1.3–1.6)	1.9 (1.8–2.1)	3.1 (2.8–3.3)
Heavy drinker	1.3 (1.2–1.5)	1.6 (1.4–1.8)	1.8 (1.6–2.0)
**Socioeconomic challenge**			
Less than high school education	1.0 (0.9–1.1)	1.1 (1.0–1.2)	1.4 (1.3–1.6)
Unemployment	1.1 (0.9–1.3)	1.3 (1.2–1.5)	1.7 (1.5–2.0)
No health insurance	1.0 (0.9–1.1)	1.1 (1.0–1.2)	1.3 (1.2–1.5)

**Abbreviation:** CI = confidence interval.

* Based on the number of adverse childhood experience types reported.

^†^ Referent group had zero adverse childhood experiences; all models were adjusted for sex, age group, and race/ethnicity.

^§^ States with state-added adverse childhood experience questions: Alaska, Kansas, Kentucky, Maryland, Ohio, South Carolina, and Texas (2015), Arizona, Arkansas, Georgia, Louisiana, Michigan, New York, Oklahoma, Pennsylvania, and Utah (2016); Connecticut, Illinois, Iowa, Nevada, Oregon, South Dakota, Tennessee, Virginia, and Wisconsin (2017).

^¶^ Overweight: body mass index ≥25 kg/m^2^; obesity: body mass index ≥30 kg/m^2^.

The largest reductions in observed outcomes were estimated to be among the group with the most exposures (four or more types of adverse childhood experiences) across all outcomes (Table 3). The estimated overall percentage reductions in chronic health conditions associated with preventing all adverse childhood experiences ranged from 1.7% for overweight or obesity to 27.0% for COPD. Substantial reductions were also estimated for depression (44.1%), current smoking (32.9%), and heavy drinking (23.9%). The reductions in socioeconomic challenges ranged from 3.8% (lack of health insurance) to 14.9% (unemployment).

**TABLE 3:** Population attributable fractions (PAFs) for health conditions, health risk behaviors, and socioeconomic challenges, by adverse childhood experience score*^,†^ — Behavioral Risk Factor Surveillance System, 25 States,^§^ 2015–2017

Outcome	Adverse childhood experience score			Overall PAF %
	1	2–3	≥4	
	PAF %			
**Chronic condition**				
Coronary heart disease	2.6	3.4	6.6	**12.6**
Stroke	—*	5.0	9.6	**14.6**
Asthma	4.2	8.1	11.7	**24.0**
Chronic obstructive pulmonary disease	4.1	9.1	13.8	**27.0**
Cancer (excluding skin)	—	2.4	3.5	**5.9**
Kidney disease	3.7	5.5	6.5	**15.7**
Diabetes	—	2.2	3.5	**5.7**
Overweight or obesity^¶^	—	0.7	1.0	**1.7**
**Mental health**				
Depression	6.4	14.7	23.0	**44.1**
**Health risk behavior**				
Current smoker	5.9	11.1	15.9	**32.9**
Heavy drinker	5.6	9.0	9.3	**23.9**
**Socioeconomic challenge**				
Less than high school education	—	—	4.6	**4.6**
Unemployment	—	5.7	9.2	**14.9**
No health insurance	—	—	3.8	**3.8**

* Adverse childhood experience categories that were not statistically different from the unexposed (zero adverse childhood experiences) group were not included in the PAF calculation and are indicated by a dash. All models were adjusted for sex, age group, and race/ethnicity.

^†^ Based on the number of adverse childhood experience types reported.

^§^ States with state-added adverse childhood experience questions: Alaska, Kansas, Kentucky, Maryland, Ohio, South Carolina, and Texas (2015), Arizona, Arkansas, Georgia, Louisiana, Michigan, New York, Oklahoma, Pennsylvania, and Utah (2016); Connecticut, Illinois, Iowa, Nevada, Oregon, South Dakota, Tennessee, Virginia, and Wisconsin (2017).

^¶^ Overweight: body mass index ≥25 kg/m^2^; obesity: body mass index ≥30 kg/m^2^.

## Discussion

Approximately three fifths of the adults among the 25-state study population experienced at least one type of adverse childhood experience, and approximately one in six reported experiencing four or more types of adverse childhood experiences. This study found that adverse childhood experiences are associated with leading causes of morbidity and mortality and with poor socioeconomic outcomes in adulthood. Persons reporting more types of adverse childhood experiences were at highest risk. These findings are consistent with those from similar analyses conducted in England, Europe, and North America (*1*,*2*) and suggest that preventing adverse childhood experiences might reduce occurrences of the outcomes examined, with potential reductions ranging from 1.7% (overweight or obesity) to 44.1% (depression). Given these findings, preventing adverse childhood experiences could have broad positive health, social, and economic impacts. For example, preventing adverse childhood experiences could potentially reduce the number of persons with coronary heart disease, the leading cause of death in the United States (*13*), by up to 12.6%, representing a potential reduction of approximately 1.1 million cases of coronary heart disease for the 25 states analyzed. Applied to national estimates in 2017, this translates to up to 1.9 million cases of coronary heart disease, 2.5 million cases of overweight or obesity, 1.5 million incidences of high school noncompletion, and 21 million cases of depression that would have been potentially avoided by preventing adverse childhood experiences (*19*).

Those who experienced four or more types of adverse childhood experiences accounted for a disproportionate share of the preventable fraction of every health and socioeconomic outcome measured. Although the prevalence of any type of adverse childhood experience was similar among men and women, the prevalence of four or more types of adverse childhood experiences was higher among women. The prevalence of adverse childhood experiences was also higher among persons aged 18–24 and 25–34 years, particularly the prevalence of four or more types of adverse childhood experiences, compared with other age groups. The higher risk among the younger groups could be due to differences across cohorts in risk, willingness to disclose, or ability to recall adverse childhood experiences. Increased mortality among those with higher adverse childhood experiences could also contribute to this pattern. Strategies to prevent adverse childhood experiences in the first place and to intervene with those who have been exposed to adverse childhood experiences might help to reduce prevalence of engaging in health risk behaviors in young adulthood and subsequent negative health outcomes. These strategies might also help to break the multigenerational cycle of adverse childhood experiences as these age groups are most likely to start families or raise children. Significant racial/ethnic inequities were also observed: AI/AN, blacks, and the Other racial/ethnic groups had substantially higher prevalences of four or more types of adverse childhood experiences, compared with whites. Communities could focus on reducing stressors these groups might face from living in underresourced neighborhoods and from historical and ongoing trauma caused by systemic racism or multigenerational poverty resulting from limited educational and economic opportunities (*14*).

Depression, heavy drinking, smoking, lower educational attainment, lack of health insurance, and unemployment were significantly associated with adverse childhood experiences. Previous research has also documented the connection between adverse childhood experiences and substance use and suicide (*6*), underscoring the importance of preventing adverse childhood experiences as a strategy for addressing the opioid overdose crisis, reducing the prevalence of suicide, and preventing leading causes of death in the United States. Prevention of adverse childhood experiences is possible with state and community efforts to build resilient families and communities, provide parental support to develop positive parenting and coping skills, and increase access to, and use of, comprehensive health services (*14*,*15*).

The findings of this report are subject to at least six limitations. First, recall and social desirability biases might reduce accuracy of self-reported adverse childhood experiences, thereby underestimating the actual prevalence of adverse childhood experiences. Second, causality cannot be inferred from these cross-sectional data. Third, data were from 25 states and might not be generalizable to other states. Fourth, the data do not assess severity, frequency, or duration of adverse childhood experiences, nor do they contrast the effects of specific types of adverse childhood experiences. Fifth, it was not possible to control for factors that could affect both adverse childhood experiences and selected outcomes (e.g., family socioeconomic position during childhood). Finally, the BRFSS adverse childhood experience module is a brief public health surveillance instrument. As such, it identifies a limited set of adverse childhood experiences and not the full range of childhood adversities. Despite these limitations, the findings from this study can help multiple sectors, including clinicians, researchers, policymakers, and the public, appreciate the connections between cumulative exposure to adversity and mental, physical, and socioeconomic outcomes.

Fundamental to adverse childhood experience prevention is the creation of safe, stable, nurturing relationships and environments for all children and families. CDC’s comprehensive approach to preventing adverse childhood experiences uses multiple strategies derived from the best available evidence (*14*). These strategies emphasize early prevention and include 1) strengthening economic supports for families (e.g., earned income tax credits, family-friendly work policies); 2) promoting social norms that protect against violence and adversity (e.g., public education campaigns to support parents and positive parenting, bystander approaches to support healthy relationship behaviors); 3) ensuring a strong start for children (e.g., early childhood home visitation, high quality child care, preschool enrichment programs); 4) enhancing skills to help parents and youths handle stress, manage emotions, and tackle everyday challenges (e.g., social emotional learning programs, safe dating and healthy relationship skill programs, parenting skill and family relationship approaches); 5) connecting youths to caring adults and activities (e.g., mentoring and after school programs). The sixth strategy is intervening to lessen immediate and long-term harms through enhanced primary care to identify and address adverse childhood experience exposures with screening, referral, and support; victim-centered services; and advancement of trauma-informed care for children, youths, and adults with a history of adverse childhood experience exposures. This is important for reducing the consequences of adverse childhood experiences and for helping to protect the next generation of children from exposure to violence and other adverse experiences, such as witnessing substance misuse in their household. Multiple studies have documented that substantial reductions in adverse childhood experiences are possible and can have broad and sustained benefits (*14*–*16*). For example, adverse childhood experience prevention strategies are associated with higher academic achievement and reductions in depression, suicidal behavior, arrest and incarceration rates, and substance use in adolescence and adulthood (*14*).

Adverse childhood experiences can contribute to a large public health burden across multiple outcomes. Effective, comprehensive approaches to preventing adverse childhood experiences are available. States and communities can use data and resources such as CDC’s Preventing Adverse Childhood Experiences (ACEs): Leveraging the Best Available Evidence (*14*) to better understand adverse childhood experiences in their locales, prioritize adverse childhood experience prevention, and improve the mental, physical, and social well-being of their populations over the lifespan (*14*).

**Summary**

**What is already known about this topic?**

Adverse childhood experiences are common and are associated with many poor health and life outcomes in adulthood.

**What is added by this report?**

Nearly 16% of adults in the study population reported four or more types of adverse childhood experiences, which were significantly associated with poorer health outcomes, health risk behaviors, and socioeconomic challenges. Population attributable fractions representing potential percentage reductions in outcomes ranged from 1.7% for overweight or obesity to 44.1% for depression.

**What are the implications for public health practice?**

Using the best available evidence to create safe, stable, nurturing relationships and environments can prevent adverse childhood experiences and could potentially prevent adult chronic conditions, depression, health risk behaviors, and negative socioeconomic outcomes.

## Footnotes

* https://www.cdc.gov/brfss.

† In 2015, data were analyzed from Alaska, Kansas, Kentucky, Maryland, Ohio, South Carolina, and Texas; in 2016, from Arizona, Arkansas, Georgia, Louisiana, Michigan, New York, Oklahoma, Pennsylvania, and Utah; and in 2017, from Connecticut, Illinois, Iowa, Nevada, Oregon, South Dakota, Tennessee, Virginia, and Wisconsin.

§ The Other race/ethnicity category consisted of participants self-reporting as non-Hispanic Native Hawaiian or Other Pacific Islander, non-Hispanic multiracial, or non-Hispanic other.
